# The strange case of Mr. H. Starting dialysis at 90 years of age: clinical choices impact on ethical decisions

**DOI:** 10.1186/s12910-017-0219-4

**Published:** 2017-11-09

**Authors:** Giorgina Barbara Piccoli, Andreea Corina Sofronie, Jean-Philippe Coindre

**Affiliations:** 1 Department of Clinical and Biological Sciences, University of Torino, Torino, Italy; 2 Nephrology, Centre Hospitalier Le Mans, Avenue Roubillard, 72000 Le Mans, France

**Keywords:** Elderly patients, Principlism, Narrative ethics, Haemodialysis, Palliative care

## Abstract

**Background:**

Starting dialysis at an advanced age is a clinical challenge and an ethical dilemma. The advantages of starting dialysis at “extreme” ages are questionable as high dialysis-related morbidity induces a reflection on the cost- benefit ratio of this demanding and expensive treatment in a person that has a short life expectancy. Where clinical advantages are doubtful, ethical analysis can help us reach decisions and find adapted solutions.

**Case presentation:**

Mr. H is a ninety-year-old patient with end-stage kidney disease that is no longer manageable with conservative care, in spite of optimal nutritional management, good blood pressure control and strict clinical and metabolic evaluations; dialysis is the next step, but its morbidity is challenging. The case is analysed according to principlism (beneficence, non-maleficence, justice and respect for autonomy).

In the setting of care, dialysis is available without restriction; therefore the principle of justice only partially applied, in the absence of restraints on health-care expenditure. The final decision on whether or not to start dialysis rested with Mr. H (respect for autonomy). However, his choice depended on the balance between beneficence and non-maleficence. The advantages of dialysis in restoring metabolic equilibrium were clear, and the expected negative effects of dialysis were therefore decisive. Mr. H has a contraindication to peritoneal dialysis (severe arthritis impairing self-performance) and felt performing it with nursing help would be intrusive. Post dialysis fatigue, poor tolerance, hypotension and intrusiveness in daily life of haemodialysis patients are closely linked to the classic thrice-weekly, four-hour schedule. A personalized incremental dialysis approach, starting with one session per week, adapting the timing to the patient’s daily life, can limit side effects and “dialysis shock”.

**Conclusions:**

An individualized approach to complex decisions such as dialysis start can alter the delicate benefit/side-effect balance, ultimately affecting the patient’s choice, and points to a narrative, tailor-made approach as an alternative to therapeutic nihilism, in very old and fragile patients.

*“Dialysis should follow life,*

*not life dialysis”*

*Interview with Sonia Angelini,*

*on renal replacement therapy for over 30 years.*

## Background

Staring dialysis at an advanced age is not only a clinical challenge but also an ethical one [1–4]. In fact, the advantages of starting dialysis at “extreme” ages are challenged by a growing body of data that show that, in patients with high comorbidity, the benefits may be offset by high dialysis-related morbidity, thus impacting on quality of life, without an advantage for survival [5–7].

Some authors have reported that with integrated clinical management, including nutritional management and optimization of symptomatic treatment, survival rates are comparable to those obtained with dialysis [7–12]. This option is often called “palliative care” or “conservative care” and some authors maintain that it should be offered as an alternative to dialysis to fragile patients with “terminal” end-stage renal disease (ESRD) [8–14]. The results are not always easy to interpret, as the choice of palliative care may be an indication of subtle fragilities or of a nihilist, depressive attitude, both associated with lower survival. Within these limits, a recent meta-analysis found equivalent one-year survival on palliative care and on dialysis [14].

The contraposition between dialysis care and conservative care may however be less Manichean. Just as the definition of alternative and complementary or allied medicines has shifted from being opposed to one another to being integrated, maximum “conservative care”, in its semeiotic meaning of “preserving the residual kidney function” should probably be offered to all patients, in particular to those with the highest comorbidity [15–17]. Some groups, including ours, have tried to graduate the dialysis indications according to the individual’s clinical needs, in an effort to progressively integrate the residual kidney function and thereby ensure a smooth shift from pre-dialysis to dialysis care [18–30].

In this report we will analyse a clinical case, referred to as “Mr H”.

In addition to the analysis of its clinical issues, looking at a case with an eye to the four principles of principlist ethics, integrated when necessary with an individual “narrative ethics” approach, can help clinicians decide what to do and how to support patients in critical phases of their disease.

Therefore, the case of Mr. H will be discussed on the basis of the four principles, to exemplify how the modulation of clinical options can change the beneficence/non-maleficence balance, thus affecting ethical reasoning and the patient’s choice [30–33]. Differently from other recently reported cases, Mr. H received extensive counselling, and all the options, including “non dialysis” were thoroughly explained and examined; therefore we will focus our discussion on how the way dialysis is done can influence the decisional process [1, 2, 4].

Furthermore, we will discuss how combining principlism with narrative ethics can furnish practical suggestions, in the context of patient-centred, individualized medicine [33–36].

## Case presentation

The case we discuss is that of Mr. H, a 90-year-old patient with end-stage kidney disease, diagnosed as nephroangiosclerosis. This clinical diagnosis was based on a long-standing history of hypertension, the presence of diffuse signs of vascular disease, the absence of severe proteinuria, relevant haematuria or systemic symptoms suggesting a different cause of chronic kidney disease (CKD). Furthermore, up to the last CKD phase, the progression trajectory had been relatively slow, after which a relatively rapid increase in creatinine was observed in the course of 1 year (creatinine 1.52 mg/dl in February 2007, 2.5 mg/dl in November 2015, 2.7 mg/dl in January 2016, 3.1 mg/dl in March 2016).

In June 2016 Mr. H was hospitalised for a further reduction in kidney function, probably after an oligo-symptomatic infection (C-reactive protein 71 mg/l, fibrinogen: 7.44 g/l). At hospitalisation, serum creatinine had reached 7.5 mg/dl, with severe acidosis (bicarbonate 15 mEq/l) and anaemia (haemoglobin 7.9 g/dl). The possibility of a cholesterol emboli syndrome was also considered, on account of the diffuse vascular disease observed, but no eosinophilia, livedo reticularis or skin lesions were present.

At hospitalisation, the patient’s weight was 80.7 kg, for a body mass index of 30.7 Kg/m2 (height 1.62 m), with no clinical signs of malnutrition; severe arthrosis and diffuse leg hyperkeratosis were also part of the clinical picture. During hospitalisation his general metabolic balance improved, but his serum creatinine stabilised between 6.0 and 7.0 mg/dL.

At 90 years of age, Mr. H lived alone, with help from the social services for home management; he received a hot meal at noon from a retirement home (in line with the usual organization of French home support). The meal served as a basis for lunch and supper, supplemented with bread, potatoes, or cheese. He kept a garden and claimed to grow the best potatoes in the area.

While the usual prognostic indexes may not be of great help in patients in advanced old age, his Charlson index was 9 (high comorbidity, two-year survival probability of 30%), i.e. a comorbidity mainly linked to age and end-stage kidney disease. Perhaps more importantly, given the close link between nutritional status and survival, his SGA (subjective global assessment) score was A (well nourished) and his MIS (malnutrition inflammation score) index was 3 (optimal).

With this background, the decision on whether or not to start dialysis was discussed, including the options of peritoneal dialysis, haemodialysis and a low-protein diet. Since Mr. H suffered from severe arthrosis, which would have prevented self-performance, and did not receive nursing care, it was decided that peritoneal dialysis should not be undertaken. Haemodialysis was considered as a potential option that could be undertaken if absolutely necessary, and a low-protein diet was chosen as the best initial option. His diet was adapted to make up a 0.6–0.8 g/Kg/day moderately restricted, plant-based, but not fully vegan, low-protein diet that was supplemented with amino and ketoacids (1 Ketosteril pill for each 10 Kg of body weight), to avoid malnutrition. Given the low probability of creating a functioning arteriovenous fistula, the placement of a tunnelled catheter was decided on in case dialysis became necessary.

Close follow-up was started, with monthly or twice monthly controls; however, acidosis was very difficult to control and rapid unpredictable weight gain was observed, requiring frequent hospitalisation, in spite of good nutritional compliance, and good blood pressure control.

From a theoretical point of view, treatment should maximize survival and improve quality of life while the negative effects produced should be minimal. This was not fully clear in our patient and the medical and nursing staff was concerned about the clinical implications of dialysis start in a patient of Mr. H’s age; therefore there were further extensive discussions of treatment options, presented by the nurses and a psychologist. After this step, Mr. H, who had initially agreed to start dialysis was reported to refuse treatment, considering it incompatible with his daily routine. Subsequent conversations revealed that the underlying problem was missing his mid-day meal: “If I come to dialysis, I won’t be home at noon; and if I’m not home at noon, the people who deliver my meals will not find me, and if they do not deliver my meals, I won’t have anything to eat. I’m 90, I have severe arthrosis, and I’m no longer able to cook my meals myself. And I want to eat.”

Identification of the communication problem (presentation of a standard haemodialysis technique of 4 hours three times per week, instead of a tailored incremental approach) led to an empiric “narrative” compromise, with a prescription of 2.30–3.0 h of dialysis, one session per week, combined with a 30-min change in the delivery time of his meals. Mr. H felt that this arrangement was compatible with his needs.

At the time of the present report, Mr. H is alive and in good clinical condition. He is still on a once-daily schedule. Some relevant biochemical data are reported in Table 1. They show good overall clinical and biochemical stability, which would probably have been difficult to achieve without treatment: even if the formulae for e-GFR calculation are not precise in advanced old age, 9 mg/dL of serum creatinine corresponds to 5–6 mL/min of GFR according to the formulae employed, and the pre-dialysis level remains quite stable, thus suggesting that we were not dealing with an acute and reversible kidney injury on the basis of a chronic renal disease (Table 1).

**Table 1:** Clinical and biochemical data before and after dialysis start

	Predialysis (last biochemical data)	1 month of HD	6 months of HD	12 months of HD
Hospitalisations	23/06/2016 to 08/07/2016	none	none	none
	08/08/2016 to 13/08/2016			
	03/10/2016 to 7/10/16			
	24/10/2016 to 01/11/2016 (dialysis start)			
Autonomy	Preserved	Preserved	Preserved	Preserved
Oedema (clinical)	Yes	No Weight loss on dialysis 0.5–1 kg	No Weight loss on dialysis 1–1.5 kg	No Weight loss on dialysis 0.5–1 kg
BP control	Difficult (160–170/ 90–100 mmHg)	OK (pre: 130–150/ 70–90-mmHg)	OK (pre: 130–150/ 70–90-mmHg)	OK (pre: 130–150/ 70–90-mmHg)
BMI (Kg/m2)	31.7	33.09	33.29	33.71
BUN mg/dL	122.4	86.24 pre	96.88 pre	113.96 pre
		34.72 post	41.16 post	50.68 post
Albumin g/dL	3.5	3.2 pre	3.6 pre	3.3 pre
		3.3 post	4.0 post	3.6 post
Haemoglobin g/dL	13.4	11.5	11	11.1
Bicarbonate mmol/L	18	24 pre	27 pre	27 pre
		26 post	28 post	22 post
Creatinine mg/dL	9.45	6.32 pre	5.71 pre	8.09 pre
		3.05 post	3.35 post	3.72 post

*BMI* Body mass Index, *BUN* Blood Urea Nitrogen, *BP* Blood Pressure

The main electrolytes are normal in the predialysis phase, except for mild acidosis (Bicarbonate between 17 and 20 mEq/L); parathyroid hormone is in the 100–300 ng/mL range and brain natriuretic peptide is between 80 and 120 pg/ml, in line with the absence of fluid overload (little need for weight loss in dialysis, on furosemide 120 mg/day). Haemoglobin has been kept on target (10–11 g/l) with recombinant erythropoietin; blood pressure has been controlled with Lercanidipine 20 mg, Nebivolol 5 mg and Urapidil 120 mg. Serum albumin is well preserved, and the patient’s “dry weight” increased after the start of dialysis. Mr. H is still on a 0.8 g of protein/Kg/day diet, supplemented with Ketosteril (1 each 10 Kg of BW).

Meanwhile, he has celebrated his ninety-first birthday. His good clinical and psychological adaptation to dialysis treatment have made the medical and nursing group confident that it will be possible to extend this option to a twice weekly schedule, with limited dialysis time (2.5–3 h) if necessary in the future.

## The principlist approach

In this first part of the discussion we analyse the case of our ninety-year-old patient on the basis of the four principles of justice, autonomy, beneficence and non-maleficence, as an integration to the clinical discussion and as a way to highlight the points in which a flexible clinical approach, in line with personalized medicine, can support a patient’s decisions.

### Justice

There are several ways to consider justice from an ethical point of view in medicine; although they are all legitimate, they differ markedly [37–44].

A “social” reading of justice sees it as the fair distribution of opportunities and resources between individuals, with particular attention to competing situations, in which the choice of care for one patient may mean a lack of availability of care for another [38–44].

Since in the setting of in which Mr. H received care (France) dialysis treatment is available without limitation for all patients needing it, we considered that he had the right to be treated. While the risk of over-treating and of a futile use of resources persists, our patient is not the only nonagenarian on dialysis, nor the oldest one reported in the literature, in which positive results have been recorded, challenging an age-centred policy [45–48].

On a more general basis, our case elicits somewhat contrasting reflections on the complex relationship between physicians’ time, delivery of resources and the cost efficiency of health care services. In this context, justice may have a more pragmatic connotation. In settings of limited resources, in which dialysis treatment is not freely available, or is limited by logistic reasons, a lower number of dialysis sessions per patient makes it possible to treat more individuals, and optimise access to care. In contrast, in high-resource settings, in which dialysis is a well-established right, and often a quite cost-efficient treatment, a flexible approach to dialysis sessions is a departure from a rigid “thrice-weekly” organisation, thus reducing the economic efficiency of the system. A tailor-made dialysis system is, of course, not incompatible with economic efficiency, but as a rule, flexibility and cost-cutting are inversely related, and treatments that differ from a time-honoured routine usually require additional control, which is obviously more demanding.

In this regard, personalized medicine, increasingly advocated as the best model of care, emphasizes the ethical issues physicians face: is their primary responsibility to guarantee the individual patient’s rights, or to ensure the economic efficiency of the health care system?

On an organizational basis, we need to take into account that personalization requires medical time, as does discussion; while the temptation to dismiss this issue by arguing that “time constraints make it impossible to follow all patients in this way” is evident, personalized medicine offers a precious tool not only for defending the autonomy of physicians’ choices, but also for ensuring that time is available for attaining this qualitative goal.

A “legal” interpretation of the sense of justice sees it as synonymous with laws. In this context, Mr. H’s right to treatment is guaranteed by French law, as is his decision not to undertake dialysis treatment if he does not wish to [49].

In addition, justice can be seen as a moral/ethical right, taking religious beliefs into account. In this regard, Mr. H’s religious beliefs may have influenced his decision on whether or not to undertake a potentially complex treatment, but once more, the decision was his, and offering dialysis to an elderly, independent and competent person does not challenge current ethical or moral norms [50–53].

### Respect for autonomy

In western countries, respect for the patient’s autonomy is usually considered to be the most important of the four principles, which is of particular importance when different principles are in conflict or are not syntonic, as in the case of Mr. H, in which the balance between beneficence and non-maleficence is particularly delicate [54, 55].

In fact, in the absence of an a priori decision in favour or against starting dialysis, the choice Mr. H. made depended on how he saw the balance between beneficence and non-maleficence, the latter consisting in the risks and side effects involved in dialysis.

A discussion of the impossibility of offering patients wholly unbiased information, as it is inevitable that physicians’ or nurses’ convictions will influence how they explain options to patients, and of the role of the “way we say” things, whose importance sometimes overrides the meaning of the content, is beyond the scope of this paper [56–58].

Information depends upon the model of care: paternalistic approaches “manoeuvre” patients towards the physician’s opinions, and can be considered intrusive, while a purely informative model often lacks empathy and participation [57–65]. Personalizing medical care goes along with a holistic approach and with an individual patient-physician relationship. This model involves presenting information honestly but not necessarily impartially, an approach in which personal opinions are mitigated, discussed and critically reviewed. In this regard, the physician counsellor’s role is to facilitate the expression of the patient’s opinions, within an empathic relationship [66–72].

By definition, empathy cannot exist without pathos, nor can counselling without convictions.

In the case of Mr. H, we chose the dynamic interaction of therapeutic alliance: in this model, the physician not only offers a choice of all the feasible options (in this case, starting dialysis or continuing with supportive treatment), but discusses with the patient how to adapt the options to their needs and preferences. When using this model, the strategy chosen may be a compromise between the “best”, or most validated option, and a reasonable, but more feasible one.

In the case of incremental dialysis, there is wide variability between specific modalities: while some authors hold that dialysis should be started with “soft” once-weekly sessions, others prefer a longer session, or define twice-weekly dialysis as incremental. In the absence of a validated, uniform way to perform this schedule, we adapted the initial policy to the patient’s needs and concerns (being home to receive meals). While in his case the initial policy proved to be successful and the original schedule was followed for 1 year, the same dynamic approach could have been followed if additional adaptations or changes had been needed.

A further issue, that here is mentioned only in passing, on account of its extreme complexity, is the feasibility of a non-experiential choice in the exquisitely subjective field of “tolerance”, in which an individual’s response is nearly impossible to foresee a priori.

In this regard, a shared decision process also supports starting dialysis on a “trial basis”, to test tolerance, and clinical advantages; no approach is without cost, but since the patient (see Table 1) had frequently been hospitalised, and was in an unstable metabolic balance, we considered that the risks of a dialysis trial (tunnelled catheter and dialysis start) were balanced by the expected benefit of improved metabolic stability.

The further implications regarding the beneficence/non-maleficence balance will be discussed below.

### Beneficence

The potential benefit of correcting the most troublesome metabolic derangements of severe CKD is obvious. In the case of Mr. H, whose glomerular filtration rate was around 5 mL/min, the presence of severe acidosis and the difficulty involved in managing the delicate balance between dehydration and fluid overload, were clear indications of the need for dialysis start.

Furthermore, while recent data challenge the policy of starting dialysis on the sole basis of a decreased glomerular filtration rate, whose reliability is uncertain in elderly patients, there is no doubt that dialysis can be successful in octogenarians and nonagenarians, especially if adapted to the needs and problems of this fragile population [73–76].

As will be further discussed, the problem may not be starting dialysis, but which dialysis should be started to maximize benefits, considering the patient’s age and life expectancy.

The potential relationship between dialysis schedule, beneficence, and non-maleficence will be discussed in detail in the next section.

### Non-maleficence

Dialysis is a prison: this harsh statement, recently published in a widely-read French newspaper, reflects the common opinion of this life-saving treatment [77]. There are several reasons why this statement may apply to dialysis patients; however, at least some of the often-cited tortures dialysis entails can be mitigated by a personalized schedule of incremental treatment.

In particular, post-dialysis fatigue is closely related to intradialytic shifts and intradialytic weight loss. The experience with short daily dialysis sessions shows that a shorter session is usually better tolerated, with an significant reduction in post-dialysis fatigue [18–25, 78–82].

If dialysis is highly intrusive in daily life, this is obviously also related to the number of sessions per week. While there is still no agreement on the definition of “incremental” dialysis, some U.S. experts presently suggest an approach based on twice-weekly dialysis [18, 19, 83–85]. In Italy, where it is often part of an integrated approach which includes nutritional management, dialysis start generally involves one session per week, while continuing the diet prescribed in the pre-dialysis phase (usually with a moderate protein restriction, at 0.6–0.8 g/Kg/day) [18–29, 86]. As Mr. H was correctly following a low-protein diet, to minimize the risk of malnutrition, we chose this second option for starting dialysis.

The slow progression of his chronic kidney disease, with good residual diuresis and lack of life-threatening episodes of ionic derangements (including hyperkalemia) further supported this approach [85–89].

No less importantly, as previously mentioned, an incremental dialysis start allowed us to perform a “dialysis test” for tolerance, and reach an experience-based decision on whether dialysis treatment should be continued, intensified or discontinued.

In our case we believe that the “treatment test” was the best way to overcome a potentially endless discussion on the advantages versus the drawbacks of the incremental dialysis policy that we had a priori identified as a clinically reasonable choice, in keeping with Mr. H’s everyday routine and quality of life.

In fact, a single 3-h dialysis session per week might not correct fluid overload or acidosis; instead a moderately protein-restricted diet (which had been prescribed in the pre-dialysis phase, and had been compatible with preserved nutritional status) could induce malnutrition. Furthermore the presence of a vascular access, or the development of intradialytic or post-dialysis hypotension could have had a negative effect on quality of life and morbidity.

The biological variability of the “old-old” is well known, and, although several experiences of incremental dialysis have been published, the data are still too scant to supply exhaustive indications (nutritional approach in well-nourished 90-year-old patient previously on a low-protein diet).

The good results obtained in our patient, apparently thanks to the combined benefits of diet and dialysis, cannot of course be taken as an a posteriori demonstration of the efficacy of this policy; conversely, they do suggest that being flexible and adapting to the patient’s needs means that an approach can be evaluated and adjusted as treatment progresses.

## The narrative analysis

While principlist ethics offer a simple framework for analysing the clinical problem, a narrative approach enables us to identify solutions in individual cases, taking into account the patient’s history, family support, fears and concerns, as well as daily life routine [33–35].

By definition, a narrativist tries to capture the stories that patients and their families tell or hide, and that, with silences as well as words, characterize decision-making [33]. Narrativism brings flexibility and personalization into the discussion and helps mitigate rigid reasoning based on the four principles, by introducing inventive solutions and practical strategies, such as changing the time of meal delivery, or scheduling dialysis sessions early to allow Mr. H to be home in time for lunch [90–92]. Furthermore, a narrative approach seeks to identify the fears or problems behind the facts, in this case, Mr. H’s fear of changing the rhythm of his daily life and losing autonomy.

The history of our patient reflects the need for a flexible approach adapted to his needs that takes into consideration the importance different individuals attribute to the constraints of daily life. A problem with the home delivery of hot meals may seem trivial in comparison to living or dying, but can appear as unsolvable to an elderly individual, becoming the focus of their fears and concerns. In this regard, finding pragmatic solutions is not only a way to come to grips with a specific inconvenience, but also shows the patient that problems can be solved and dialysis can be adapted to their needs and wishes, and need not be “a prison”.

### Combining principlism and narrative analysis

In terms of a principlist analysis, it seemed likely that Mr. H would benefit from a flexible approach to dialysis, which would serve to overcome his health problems (benefit: clinical stabilisation in the context of a uremic syndrome with difficulty in attaining a stable clinical balance with non-dialytic conservative care). Attention to minimizing side effects (non-maleficence) led to the choice of an incremental dialysis approach, in a setting in which the principle of justice was respected, at least for treatment availability (no competition with other patients who also needed dialysis). While the physicians were mainly concerned with finding the best compromise between beneficence and non-maleficence, the patient was worried about the disruption of his daily routine (home-delivered meals). While he was not a priori against dialysis (he had initially accepted it), he subsequently refused treatment because “If I’m not home… I won’t have anything to eat”.

A rigid interpretation of autonomy would probably have led to his not starting dialysis (the patient was informed, competent, and free to choose); inquiring in detail about what had made him change his mind, and asking about his habits and quality of life, identified a problem (missing meal delivery) and found pragmatic solutions that did not impact on clinical aspects, but modulated the individual’s choice (autonomy). Narrativism allowed us to get a detailed picture of the patient and mitigated the rigidity of the four principles; while many of the considerations regarding dialysis start could apply to other elderly patients with end-stage kidney disease, the patient’s choice was unique to Mr. H, as the solution also was.

In this regard, we suggest that principlism allows us to arrive at an initial analysis of difficult cases, identifying the specific aspects of the problems involved, while narration serves to put problems in perspective and possibly solve them.

## Discussion and conclusions

The case discussed illustrates the limits of a Manichean contraposition between dialysis and palliative care.

In this extreme balance, dialysis is intended as “renal replacement therapy” (RRT), a treatment that has to deliver, regardless of the residual renal function, full artificial depuration. While the definition of “adequate” and “optimal” dialysis is not clear, a strict “RRT approach” ignores residual renal function, in spite of the fact that its presence is one of the most important survival markers [18, 25–27, 87–89].

Palliative-conservative care is defined as a comprehensive treatment which allows for reasonable survival, with a good quality of life, without dialysis [10–14]. However, it is not clear why we should not offer this to all patients, and start dialysis only when an acceptable clinical balance can no longer be maintained using conservative treatment.

Indeed, this sharp opposition between palliative-conservative care and dialysis, which is often thought to be unavoidable, has begun to be seen as less of a contrast, and what is now being proposed is the integration of the two in what is called kidney supportive care, aimed at improving the quality of life, whenever possible together with therapies intended to prolong life, such as dialysis. Furthermore, the term palliative dialysis is also occasionally used to indicate a form of patient-centered dialysis that focuses on quality of life.

In our case, the decision to start dialysis was postponed until the time when “conservative care” could no longer ensure well-being. The once-weekly session was started to correct acidosis, reduce overload, and allow some degree of correction of uremic intoxication. The interventions were graduated to clinical context and life expectancy, and their purpose was to correct the derangements associated with short-term clinical problems (acidosis and overload).

The contraposition between RRT and palliative-conservative care points to what is now called “transition” to dialysis [11–14]. Studies on the high risk of death during the first months of dialysis deal with an abrupt transition from pre-dialysis care to thrice weekly 4-h haemodialysis sessions (or to a “full” schedule on peritoneal dialysis).

The choice of starting a “full schedule” of dialysis is often the only one possible in patients with minimal or no predialysis follow-up that start dialysis when admitted to the emergency room. However, in patients who have been consistently followed, the best choice may be to progressively increase their scheduled visits, from every 3 months to once a month to weekly, to a once-weekly dialysis sessions.

The case of Mr. H, in which the availability of a “soft” dialysis start smoothed the terms of a tough ethical dilemma, without forcing us to sacrifice quality to quantity of life, is a story with a happy ending, at least for the time being: our patient is doing well on dialysis, enjoys his role as our “oldest patient”, gets home by the time his meal is delivered, and tolerates dialysis sessions well.

Concerned about his daily routine, Mr. H would not have accepted more intrusive treatment which, as his story shows, was not really needed; his positive experience with dialysis will modulate his further choices, including intensifying dialysis, if need be.

In conclusion, this case encourages us to reflect on the reasons why laypeople and many physicians have a negative view of dialysis. Is dialysis in itself a killer, or is the way we use this treatment the cause of “dialysis shock”?

Unlike standardized medicine, based on identifying the best solution, in an ethical discussion there is no best solution, but instead a personalized choice adapted to the context and to the individual. As in chemotherapy, in which protocols are increasingly being adapted to patients, there is room and a need for well-grounded “personalized medicine” in renal replacement therapy.

## Abbreviations

CKD: Chronic kidney disease
ESRD: End stage renal disease
RRT: Renal replacement therapy
